# Rhubarb-Aconite Decoction (RAD) Drug-Containing Serum Alleviated Endotoxin-Induced Oxidative Stress Injury and Inflammatory Response in Caco-2 Cells In Vitro

**DOI:** 10.1155/2020/5834502

**Published:** 2020-07-04

**Authors:** Xiao-hong Du, Qing-jun Chen, Jian-bo Song, Yan Xie, Yan Zhi, Ru-ru Sun, Guo-hui Liu, Xin Kang

**Affiliations:** ^1^Department of Hepatobiliary Pancreatic Surgery, First Hospital Bethune of Jilin University, Changchun 130021, China; ^2^Department of Emergency Medicine, Beijing Hepingli Hospital, Beijing 100013, China; ^3^Department of Emergency Medicine, Affiliated Zhongshan Hospital, Dalian University, Dalian 116001, China; ^4^Organ Transplant Center, Tianjin First Central Hospital, Tianjin 300000, China; ^5^Department of Burns, The Second Affiliated Hospital of Kunming Medical University, Kunming 650101, China; ^6^Department of Emergency Medicine, The First Hospital of Jilin University, Changchun 130012, China

## Abstract

Rhubarb-Aconite Decoction (RAD), a famous Chinese medicine prescription, has been widely used for treating intestinal injury. However, the effect of RAD on intestinal epithelial cells is unclear. The aim of this study was to investigate the effects of RAD drug-containing serum on the oxidative stress injury and inflammatory response induced by endotoxin (ET) in Caco-2 cells in vitro. Lipid peroxide malondialdehyde (MDA), lactate dehydrogenase (LDH), caspase-11, tumor necrosis factor-*α*(TNF-*α*), interleukin-3(IL-3), and cytokeratin (CK)18, adenosine triphosphate (ATP) activity, and intracellular free calcium ion levels were measured. The results showed that ET triggered the activation of caspase-11 and the massive release of TNF-*α*, increased the inhibitory rate of cell growth, MDA, and LDH expressions in Caco-2 cells. Moreover, RAD drug-containing serum could inhibit caspase-11 activation, decrease the release of TNF-*α* and IL-3, reduce intracellular free calcium ion, and enhance CK 18 expression and ATP activity. These novel findings demonstrated that ET-induced oxidative stress injury and inflammatory response of Caco-2 cells were improved by RAD drug-containing serum, indicating that RAD may be a good choice for the treatment of intestinal injury.

## 1. Introduction

The intestine is the core organ of posttraumatic stress and the initiating organ of multiple organ dysfunction in the development of severe complications under critically stressful events [1–3], including trauma [4], burns [5], and brain injury [6, 7]. These stressful events may initiate a cascade of intestinal events, including the destruction of the intestinal mucosa, barrier dysfunction, translocation of intestinal bacteria, and endotoxin (ET), a major component of the outer membrane of Gram-negative bacteria, which may cause systemic inflammatory response syndrome (SIRS) and multiple organ dysfunction syndrome (MODS) [8]. The intestinal epithelial is known as the most critical barrier for protection against harmful antigens and pathogens [9]. Oxidative stress injury and inflammatory response have been implicated in the dysfunction of the intestine barrier. It is necessary to reduce oxidative stress injury and inhibit the inflammatory response to protect the normal structure and function of intestinal epithelial cells. At present, people often use chemical methods to synthesize antioxidants, but animal experiments show that they have certain toxicity and carcinogenic effects [10–12]. Thus, natural antioxidants are urgently needed, and Traditional Chinese medicine (TCM) is deemed promising to avoid the oxidative injury of intestine.

Rhubarb-Aconite Decoction (RAD), a famous Chinese medicine prescription, was originally described in Chinese Medical Classics-Jin Kui Yao Lue. RAD consists of Radix et Rhizoma Rhei, Radix Aconiti Lateralis Praeparata, and Radix et Rhizoma Asari, which has been widely used for intestinal obstruction, chronic diarrhea, and intestinal injury [13–15]. The study showed that RAD reduced serum ET level, stimulated intestinal peristalsis, and protected intestinal mucosal barrier function in patients with severe acute pancreatitis [16, 17]. However, the effect of RAD on the role of intestinal epithelial cells is unclear. The serum pharmacological method of TCM, a semidetached residential in vivo experiment method, has obvious advantages in the study of the pharmacological effects of TCM [18, 19]. Serum pharmacological methods may contribute to the study of the effects of RAD on intestinal epithelial cells in vitro. So, the aim of this study was to investigate the effects of RAD drug-containing serum on the oxidative stress injury and inflammatory response induced by ET in human Caco-2 cells in vitro.

## 2. Materials and Methods

### 2.1. Materials and Reagents

Human colon Caco-2 cells were obtained from the American Type Culture Collection (Rockville, MD, US). Anti-CK 18 antibody was supplied by Abcam plc (Cambridge, UK). Dulbecco's modified eagle medium (DMEM), fetal bovine serum (FBS), L-glutamine, nonessential amino acids, penicillin, and streptomycin were purchased from Life Technologies (Carlsbad, CA, USA). ET derived from *Escherichia coli* 0127:B8 and Thiazolyl Blue tetrazolium bromide (MTT) were all from Sigma (St. Louis, MO, the United States). Malondialdehyde (MDA), Adenosine triphosphate (ATP), and lactate dehydrogenase (LDH) assay kits were obtained from Nanjing Jiancheng Bioengineering Institute (Nanjing, Jiangsu, China). Enzyme-linked immunosorbent assay (ELISA) kits and Western blot kits of caspase-11, tumor necrosis factor *α* (TNF-*α*), and interleukin 3 (IL-3) were from Elabscience Biotechnology Co.Ltd (Elabscience, Wuhan, China) and Biovision Co.Ltd (San Francisco, CA, the United States). BCA Protein Assay Kit was obtained from KeyGen Biotech. Co. Ltd (Nanjing, Jiangsu, China). Ponceau S was purchased from Puhe Biological Medicine Technology Co., Ltd (Wuxi, Jiangsu, China). Fluo-3-am was obtained from Life Technologies Corporation (Paisley, UK). Tween-20 was obtained from AMRESCO, Ltd. (Solon, OH, the United States) and OsO4 from Amresco, Ohio, the United States. All other chemicals and reagents used were of analytical grade.

### 2.2. Preparation of RAD Drug-Containing Serum

#### 2.2.1. Animals

Six SD rats (aged eight weeks, weighted 250 ± 20 g) were provided by the Dalian Medical University Experimental Animal Center (Dalian, Liaoning, China), which were housed in the institutional animal facility with standard animal room conditions (25°C, 12-hour light-dark cycles, ≤3 animals in a cage). All animals used in this study were cared for in compliance with the Guide for the Care and Use of Laboratory Animals. All experimental procedures using live animals were approved by the Animal Research Ethics Committee of Affiliated Zhongshan Hospital of Dalian University. Anesthetic drugs and all other necessary measures were used to reduce animal suffering during experimental procedures.

#### 2.2.2. Serum Preparation

RAD is composed of 3 species of herbal plants (rhubarb, aconite, and Asarum), each dried crude drug of which were purchased from Tong Ren Tang Group Co., Ltd (Beijing, China). Rhubarb (9g), aconite (9g), and Asarum (3g) were mixed in the ration of 3 : 3:1 (w/w). First, aconite was soaked in water (1 : 25) for 30 mins, followed by extraction in boiling water (100°C) for 1 h. Then, rhubarb was added and boiled for 10 min. Finally, Asarum was added and boiled for 5 mins, and RAD was obtained.

RAD drug-containing serum was prepared by serum pharmacological method [19, 20]. SD rats were divided into two groups for preparing RAD drug-containing serum and blank serum, respectively. One group of rats (*n* = 3) were given RAD through gavage at the doses of 20 g/(kg·d) for three days. After 2 h of the last treatment, abdominal aortic blood was collected under aseptic condition and then centrifuged at 3,000 r/min for 15 mins to obtain RAD drug-containing serum (RAD-CS). The other group of rats (*n* = 3) were given normal saline, and then obtained blank serum (BS). All serum was filtered through 0.22 *μ*m filter membrane and inactivated by water bath at 56°C for 30 mins, and then stored at –20°C until being used for the pharmacological study.

### 2.3. Cell Culture, Passage, and Grouping

**Figure 1:**
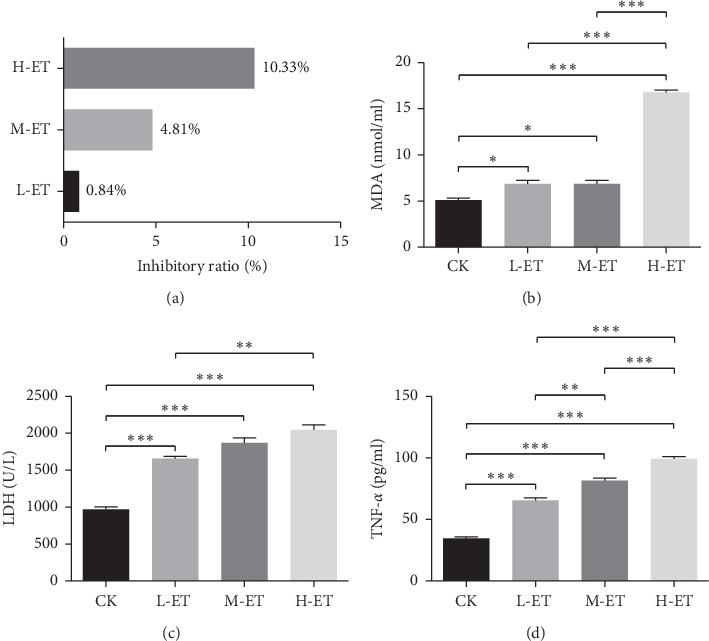
Effects of different doses of endotoxin (ET) on the inhibitory ratio of cell growth, malondialdehyde (MDA), Lactate dehydrogenase (LDH), and tumor necrosis factor-*α* (TNF-*α*) levels of Caco-2 cells. CK group: Caco-2 cells, L-ET group: Caco-2 cells stimulated with Low-dose ET (0.1 EIU/ml), M-ET group: Caco-2 cells stimulated with medium-dose ET (1.0 EIU/ml), H-ET group: Caco-2 cells stimulated high-dose ET (10.0 EIU/ml). Graphs show mean ± SD. Based on one-way ANOVA statistical analysis. ^*∗*^*P* < 0.05, ^*∗∗*^*P* < 0.01, ^*∗∗∗*^*P* < 0.001.

Caco-2 cells were cultured in DMEM medium supplemented with 10% heat-inactivated FBS, 4.0 mM L-glutamine, 1% nonessential amino acids, 100 U/ml penicillin, and 100 *μ*g/mL streptomycin, and maintained in an incubator with 5% CO_2_ humidified at 37°C. When the degree of cell confluence reached 80%, cell passage was carried out. The medium was changed every other day until the cells were fully differentiated. Caco-2 cells between the 50th to 60th passages were seeded on the apical compartment of Transwell® plates at a density of 1 × 10^4^ cells/well and were inoculated in an incubator with 5% CO_2_ at 37°C for 24 h. Then, the experiments were conducted with 4 groups: CK group (only Caco-2 cells), Low-dose ET group (L-ET), Medium-dose ET group (M-ET), and high-dose ET group (H-ET). CK group cells were not stimulated with ET. The Caco-2 cells in the L-ET group, M-ET group, and H-ET group were treated with 0.1, 1.0, and 10.0 EIU/ml of ET, respectively. Cells of each group were transferred to the Petri plate and cultured together with ET for 48 hours, and then inhibitory rate, MDA, LDH, and TNF-*α* of cells treated with ET were detected according to the kit instructions. In addition, mitochondrial structure was observed under transmission electron microscope (TEM). The results of these indicators are shown in Figure 1, which proved that 10.0 EIU/ml of ET significantly induced inflammatory response and oxidative stress injury of Caco-2 cells, and the cells stimulated with 10.0 EIU/ml of ET were used in subsequent experiments.

### 2.4. The Effect of RAD Containing Serum on Caco-2 Cells Stimulated by ET

#### 2.4.1. Cells Treatment

To investigate the effect of RAD containing serum (RAD-CS) and blank serum (BS) on 10.0 EIU/ml ET-induced inflammatory and oxidative stress injury of Caco-2 cells, we took Caco-2 cells for the following experiment. These cells were divided into four groups: CK, ET, ET + BS, and ET + RAD-CS groups. Caco-2 cells in the ET group were stimulated with 10 EIU/ml of ET. ET + BS group cells were stimulated with 10 EIU/ml of ET and then cocultured with BS. ET + RAD-CS group cells were stimulated by 10 EIU/ml of ET and then cocultured with RAD-CS. The CK group cells did not have any treatment. These cells were cultured with DMEM that changed once a day, until the growth of cells filled with dish. Then, cell inhibitory ratio, antioxidant activity, inflammatory cytokines, and mitochondrial morphology changes were measured.

#### 2.4.2. MTT Evaluated Inhibitory Ratio of Cells

The inhibitory ratio of Caco-2 cells was evaluated by MTT Cell Proliferation and Cytotoxicity Assay Kit. The concentration of Caco-2 cells was adjusted by 1 × 10^5^ cells/ml, then seeded and cultivated in 96 well culture plate (100ul each hole) at 37°C in an incubator with 5% CO_2_ for 24 h for detecting cell inhibitory ratio by MTT. The optical density (OD) value was measured by a microplate reader (Multiskan MK3, Thermo, United State) at 492 nm wavelength. Inhibitory ratio (%) = (1 − average OD treatment group/average OD CK group) × 100%.

#### 2.4.3. Mitochondrial Morphology Observation by Transmission Electron Microscopy (TEM)

Caco-2 cells in each group were fixed with 0.25% glutaraldehyde (Sigma-Aldrich, MO, the United States) in 0.1 M cacodylate buffer, pH 7.4, for 4 h, followed by postfixation in 2% OsO_4_ in 0.1 M cacodylate buffer for 1 h. These cells were washed in distilled water, dehydrated in a graded series of ethanol, and then substituted with acetone, dried by sublimation in Peldri II (Plano GmbH, Marburg, Germany), sputter-coated with gold, and at last observed by TEM (HT7700, Hitachi, Japan).

#### 2.4.4. Measurement of Caspase-11, TNF-*α*, and IL-3 by ELISA and Western Blot

Caspase-11, a specific sensor for intracellular ET recognition, mediates the noncanonical inflammatory pathway, which was detected by ELISA. Caco-2 cells concentration was adjusted to 1 × 10^5^ cells/ml and inoculated in a 96-well culture plate, 100 *μ*l per well, cultured in an incubator with 5% CO_2_ at 37°C for 24 h. Then, the supernatant was collected after 24 h, and caspase-11 concentration was measured at 450 nm with the microplate reader. In addition, TNF-*α* and IL-3 expressions were also measured by ELISA kits.

Western blot was used for semiquantitative determination of expression of caspase-11, TNF-*α*, and IL-3. Total proteins were extracted from Caco-2 cells and lysates were separated by Sodium Dodecyl Sulfonate-Polyacrylamide Gel Electrophoresis (SDS-PAGE). The proteins were then transferred to polyvinylidene difluoride (PVDF) membrane (Millipore, ON) for determination of specific protein expression using a Bio-Rad Imaging System (Bio-Rad Biosciences, Hercules, CA, United States). Relative expression was normalized to *β*-actin.

#### 2.4.5. LDH Measurement by Spectrophotometer

The antioxidant activity of RAD drug-containing serum on the Caco-2 cells was evaluated by detecting LDH expression by the spectrophotometer. Caco-2 cells concentration was adjusted to 1 × 10^5^ cells/ml. It was vaccinated in 24 hole culture plate, 1 ml per hole, in a 5% CO_2_ incubator at 37°C for 24 h. The cell supernatant was incubated for 24 h. The cell supernatants were detected according to LDH kit instructions and detected at 450 nm.

#### 2.4.6. Expression of CK18 by Confocal Microscope

CK 18 is a characteristic marker of intestinal epithelial cells. Caco-2 cells were adjusted to 1 × 10^5^ cells/ml, inoculated in a 24-well culture plate, 1 ml per well, incubated in a 5% CO_2_ incubator at 37°C for 24 h. The supernatant was aspirated, and cells were fixed with 4% paraformaldehyde for 10 mins. Endogenous peroxidase was inactivated. The cells were incubated with 3% H_2_O_2_/PBS for 10 mins at room temperature, rinsed with PBS for 5 mins, and repeated 3 times. The slice was immersed in an antigen retrieval solution (trisodium citrate buffer) at 96°C for 5 mins, washed with 2 ml PBS by a 1 ml pipette, repeated 3 times (5 mins each time), and then closed by 10% FBS at room temperature for 15 mins. CK18 antibody was added and incubated at 4°C overnight. The slice was washed with 2 ml PBS by a 1 ml pipette, repeated 3 times, for 5 mins each time. Secondary antibody IgG-CY3 was added to the section and incubated at 37°C for 30 mins. The slice was rinsed with PBS 3 times, 5 mins per time. Cells were stained with Hoechst for 30 mins. The expression of CK18 was observed under confocal microscopy.

#### 2.4.7. Detection of Mitochondrial ATP Concentration and Intracellular Calcium Ion by Flow Cytometry

In order to further study the effect of RAD drug-containing serum on Caco-2 cells, the changes of mitochondria ATP concentration and intracellular free calcium ion were measured by flow cytometry. After the cells in the 24-well plate were fixed with 4% paraformaldehyde, the Fluo-3-am probe was incubated for 30 mins, the flow detection was performed, and the expression of fluorescence value was measured by flow cytometry.

### 2.5. Statistical Analysis

The data were expressed as mean ± standard deviation (SD). The statistical analyses were performed with GraphPad Prism 6.0 software (San Diego, California, United States). Differences among multiple groups were assessed by one-way ANOVA. A value of *P* < 0.05 was considered statistically significant.

## 3. Results

### 3.1. ET Inhibited Growth of Caco-2 cells

In order to determine the effect of ET on Caco-2 Cells growth, we tested the inhibitory ratio of cells by MTT. As shown in Figure 1(a), the inhibitory ratio of Caco-2 cell stimulated with 0.1, 1.0, and 10.0 EIU/mL of ET were 0.84%, 4.81%, and 10.33%, respectively, which indicated that 10.0 EIU/mL of ET exerted maximum inhibitory ratio of Caco-2 cell growth.

### 3.2. ET Caused Oxidative Stress Injury and Release of Inflammatory Cytokines of Caco-2 Cells

To evaluate the oxidative stress injury of Caco-2 cells, MDA and LDH levels were measured. As shown in Figures 1(b) and 1(c), MDA and LDH levels in L-ET, M-ET, and H-ET groups were all higher than that in the CK group (*P* < 0.05), and their concentration reached the highest level after Caco-2 Cells were stimulated with 10.0 EIU/mL of ET, which indicate that ET caused obvious oxidative stress injury of Caco-2 cells. TNF-*α*, a proinflammatory factor, could promote the recruitment and activation of other inflammatory mediators. As shown in Figure 1(d), TNF-*α* in the H-ET groups was higher than that in the CK, L-ET, or M-ET groups, respectively (*P* < 0.001).

### 3.3. ET Caused Mitochondrial Injury of Caco-2 Cells

**Figure 2:**
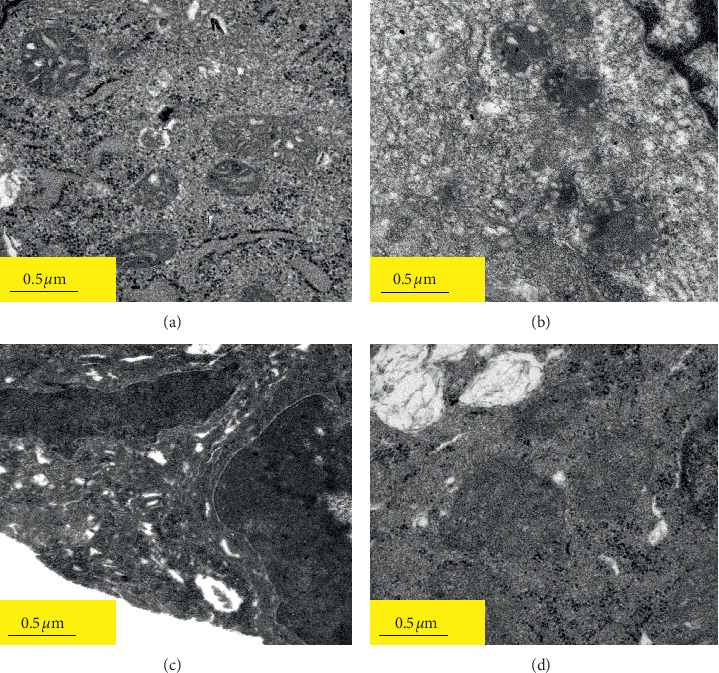
Mitochondrial changes of Caco-2 cells stimulated with different doses of endotoxin (ET) under transmission electron microscopy (TEM). (a) CK group: only Caco-2 cell, (b) L-ET group: Caco-2 cells stimulated with Low-dose ET (0.1 EIU/ml), (c) M-ET group: Caco-2 cells stimulated with medium-dose ET(1.0 EIU/ml), (d) H-ET group: Caco-2 cells stimulated high-dose ET (10.0 EIU/ml). Scale: 0.5 *μ*m. Magnifications: ×5000.

Caco-2 cells' mitochondria in the CK group were intact and its crista is distributed evenly (Figure 2(a)). Mitochondrial morphology of cells in the L-ET group and M-ET group were destroyed in different degrees (Figures 2(b) and 2(c)). In the H-ET group, the mitochondrial structure of Caco-2 cells was significantly damaged, membranes and crista severely dissolved, and their injury degree was the most serious compared with the other three groups (Figure 2(d)).

### 3.4. RAD Containing Serum Decreased the Expression of Caspase-11, TNF-*α*, and IL-3 Protein Induced by ET

**Figure 3:**
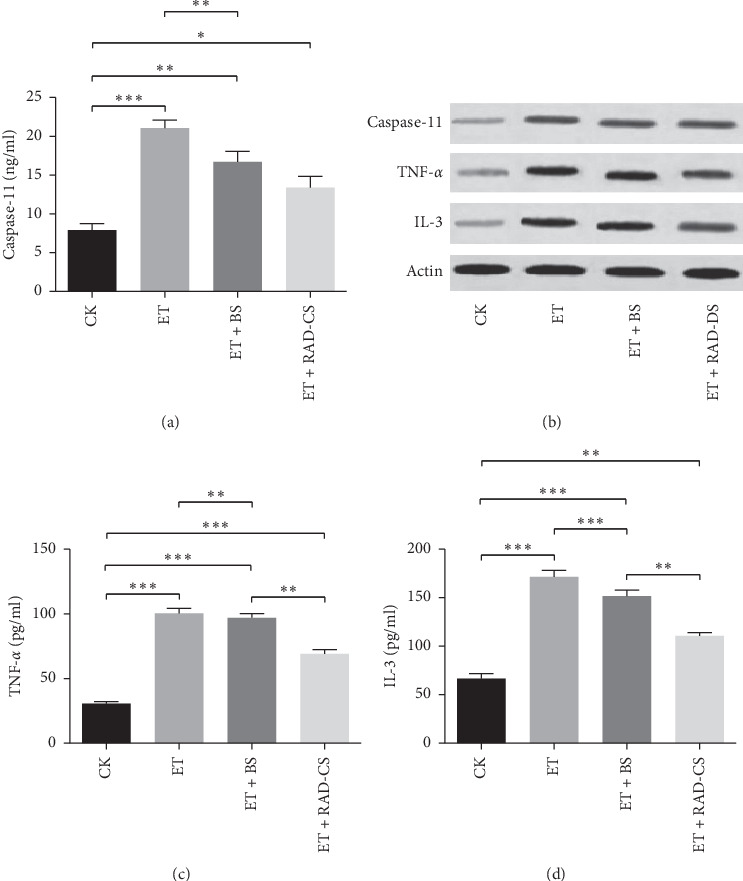
Effects of RAD containing serum on caspase-11, tumor necrosis factor-*α* (TNF-*α*), and interleukin-3 (IL-3) in Caco-2 cells induced by endotoxin (ET) by ELISA and Western blot. BS: blank serum. RAD-DS: RAD drug-containing serum. ET group: Caco-2 cells were stimulated with 10 EIU/ml of ET, ET + BS group: cells were stimulated with 10 EIU/ml of ET and then cocultured with BS, ET + RAD-CS group: cells were stimulated with 10 EIU/ml of ET and then cocultured with RAD-CS, CK group: control group. (a, c, d) based on ELISA analysis, (b) based on Western blot analysis. Graphs show mean ± SD. Based on one-way ANOVA statistical analysis. ^*∗*^*P* < 0.05, ^*∗∗*^*P* < 0.01, and ^*∗∗∗*^*P* < 0.001.

Caspase-11 plays a key role in recognition of intracellular ET. As depicted in Figure 3(a), ET dramatically increased the secretion of caspase-11 in Caco-2 cells compared with CK group, and RAD drug-containing serum inhibited significantly the activation of caspase-11 induced by ET compared with blank serum according to ELISA results. In addition, Western blot results also demonstrated that the expression of caspase-11 protein in the ET group was stronger than that of the CK group, but there was no significant difference between the ET + BS group and the ET + RAD-CS group (Figure 3(b)).

From Western blot results, the expressions of TNF-*α* and IL-3 protein in the ET group were stronger than that of CK group, and RAD containing serum reduced significantly the expression of TNF-*α* and IL-3 protein compared with the ET group and the ET + BS group (Figure 3(b)). Moreover, ELISA results also showed that RAD containing serum inhibited significantly ET-induced secretion of TNF-*α* and IL-3 (Figures 3(c) and 3(d)).

### 3.5. RAD Containing Serum Decreased LDH Levels

**Figure 4:**
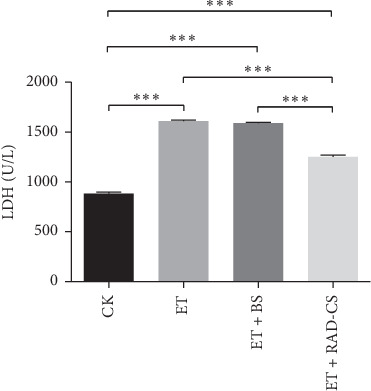
Effect of RAD drug-containing serum on lactate dehydrogenase (LDH) of Caco-2 cells induced by endotoxin in vitro. Values are mean ± SD, based on one-way ANOVA statistical analysis. ^*∗∗∗*^*P* < 0.001.

LDH levels were used to evaluate the oxidative stress injury of Caco-2 cells. As depicted in Figure 4, ET significantly increased LDH level compared with CK group (*P* < 0.001), while LDH levels in ET + RAD-DS group were higher than those in ET + BS group and ET group, which suggested that RAD containing serum could decrease LDH levels induced by ET (Figure 4).

### 3.6. RAD Containing Serum Enhanced CK18 Expression of Caco-2 Cells

**Figure 5:**
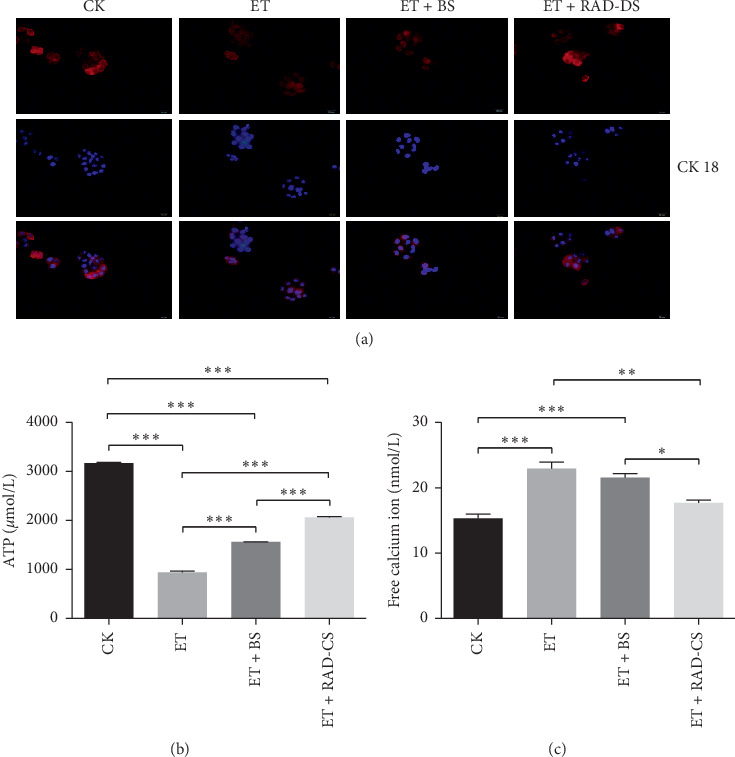
effects of RAD drug-containing serum on cytokeratin (CK)18, adenosine triphosphate (ATP), and intracellular free calcium ions in Caco-2 cells stimulated with endotoxin (ET). BS: blank serum. RAD-DS: RAD drug-containing serum. (a) Immunofluorescence staining of CK18 (red) and Hoechst staining of the nucleus (blue). (b) ATP level and (c) free calcium in groups. Values are mean ± SD. Based on one-way ANOVA statistical analysis. ^*∗*^*P* < 0.05, ^*∗∗*^*P* < 0.01, and ^*∗∗∗*^*P* < 0.001.

CK18, a marker factor of epithelial cells, was analyzed by immunofluorescence. As shown in Figure 5(a), CK18 expressions of Caco-2 cells in the CK group were the strongest among the 4 groups, and which were significantly weakened in the ET group compared with the GK group. Further, we also found that the expression of CK18 of Caco-2 cells was enhanced after the addition of RAD drug-containing serum. These immunofluorescence results suggested that RAD containing serum alleviated Caco-2 cells injury was induced by ET.

### 3.7. Effect of RAD Containing Serum on ATP and Intracellular Free Calcium Ion Levels

As shown in Figure 5(b), ATP levels in the ET group were significantly lower than that of the CK group (*P* < 0.001), ATP levels in the ET + BS group, and the ET + RAD-CS group were higher than that of the ET group (*P* < 0.001). As shown in Figure 5(c), ET increased significantly the intracellular free calcium ion level, which was reversed by RAD containing serum, while blank serum was not (*P* < 0.05).

## 4. Discussion

The major findings of our study can be summarized as follows. (1) ET triggered the activation of caspase-11 and the massive release of TNF-*α* and IL-3 increased the MDA and LDH levels, which could successfully induce an inflammatory response and oxidative stress injury of Caco-2 cells. (2) RAD drug-containing serum could alleviate ET-induced inflammatory response and oxidative stress injury of Caco-2 cells by inhibiting caspase-11 activation, the release of TNF-*α* and IL-3, and reducing intracellular free calcium ion.

The intestinal epithelium, often considered the primary defense for the host, forms a robust barrier to various infectious or noninfectious stimulations for maintaining intestinal mucosal homeostasis [21]. Oxidative stress and cytokines in the intestine in space and time orchestrate the initiation, development, exacerbation, and recurrence of the inflammatory process in various intestinal inflammatory diseases [22, 23]. ET (or lipopolysaccharides, LPS) is a component of the outer membrane of Gram-negative bacteria that live in the intestine, which could trigger oxidative stress and inflammatory response in a variety of cells [24–27]. In this study, we found that ET promoted caspase-11 activation, inhibited cell growth, increased MDA and LDH expressions, and disrupted the mitochondrial morphology, which indicated that ET led to oxidative stress injury of Caco-2 cells. In addition, the mitochondrial morphological injury of Caco-2 cells became more serious with the increase of ET dose. The serum pharmacology method of TCM is a semi in vivo experimental method in which an ancient Chinese medicine or a Chinese medicine compound is orally administered to an animal for a certain period of time, and then animal blood is collected, separating serum for preparing serum contained pharmaceutical ingredient, and in vitro experiments are performed. The serum pharmacology method provides a better way to study the effect of RAD on oxidative stress injury and inflammatory response of Caco-2 cells induced by ET in vitro. In the current study, the rats were given RAD by gavage and then the RAD containing serum was prepared by blood collection from the abdominal aorta. We took this serum to treat Caco-2 cells stimulated with ET, and then found that RAD containing serum significantly inhibited the caspase-11 activation and the release of LDH. The dynamic regulation of free calcium ions was fundamental for cell life [28]. Previous studies have shown that stress can lead to an increase in intracellular free calcium levels [29, 30]. In the present study, we found that RAD drug-containing serum could reduce cytosolic free calcium ion ([Ca2+]i) level of Caco-2 cells after being stimulated with ET. The low level of Ca^2+^ made the Ca^2+^ develop an ability as a second messenger, through the energy-consuming pumping activity of Ca^2+^ ATPases. Excess free calcium ions in cells were toxic and caused the massive activation of proteases and phospholipases. Stressful events could induce alterations in the gut mucosa, barrier function, and small intestinal motility [31]. In addition, ET could significantly decrease the CK 18 expression and ATP activity, further disrupting the structure and energy supply of Caco-2 cells, which were improved by RAD drug-containing serum. Taken together, the present study suggested that RAD drug-containing serum could significantly alleviate the ET-induced oxidative stress injury of Caco-2 cells.

To understand the underlying molecular mechanism of RAD In intestinal injury, the inflammatory reaction was analyzed by detecting the expression levels of TNF-*α* and IL-3. The intestine is a core organ where the inflammatory response occurs with the release of various inflammatory cytokines under stressful conditions, which has a potent impact on the small intestine. Especially, the inflammatory response may not only affect the intestine itself, but may also influence the function and integrity of remote organs and tissues, including the lung, liver, and brain, thus inducing SIRS and MODS. The intestine has been labeled the ‘trigger' of the inflammatory responses under severe stress, which are mainly mediated by various inflammatory cytokines [3]. In the present study, the expressions of proinflammatory cytokine TNF-*α* and IL-3 were significantly increased in Caco-2 cells stimulated with ET compared with unstimulated Caco-2 cells. Moreover, ET also inhibited the cells growth and led to mitochondrial injury of Caco-2 cells. Previous studies indicated that the release of inflammatory cytokines induces a delay in small intestinal motility and intestinal obstruction [32–34]. Moreover, inflammatory cytokines exert cytotoxic effects that induce damage to microvilli, resulting in the destruction of tight intercellular junctions and increased intestinal barrier dysfunction [35, 36]. Many studies have found that traditional Chinese medicine could reduce intestinal inflammation [37, 38]. Alabi QK reported that polyphenol-rich extract of Ocimum gratissimum leaves ameliorates colitis via attenuating colonic mucosa injury and regulating proinflammatory cytokines production and oxidative stress [39]. Our study suggested that RAD drug-containing serum significantly decreased TNF-*α* and IL-3 levels of Caco-2 cells after ET stimulation, while blank serum did not have the anti-inflammatory effect. Therefore, the intestinal inflammatory response may contribute to intestinal injury, and RAD could relieve intestinal injury by inhibiting the inflammatory response of Caco-2 cells.

## 5. Conclusions

In summary, we cultured Caco-2 cells as experimental subjects and found that ET induced the oxidative stress injury and inflammatory response of Caco-2 cells. In addition, we verified that RAD drug-containing serum alleviated ET-induced oxidative stress injury and inflammatory response of Caco-2 cells by inhibiting the activation of caspase-11 and the release of TNF-*α* and IL-3 and enhancing the antioxidant effect of cells. Although current findings are provoking, more in vivo studies are required to validate the protection effects of RAD on the intestine injury.

## Data Availability

The data used to support the findings of this study are included within the article.

## Abbreviations

RAD: Rhubarb-aconite decoction
ET: Endotoxin
MDA: Lipid peroxide malondialdehyde
LDH: Lactate dehydrogenase
CK18: Cytokeratin 18
SIRS: Systemic inflammatory response syndrome
MODS: Multiple organ dysfunction syndrome
TCM: Traditional Chinese medicine
MTT: Thiazolyl blue tetrazolium bromide
TEM: Transmission electron microscopy
SDS-PAGE: Sodium dodecyl sulfonate-polyacrylamide gel electrophoresis
PVDF: Polyvinylidene difluoride
TNF-*α*: Tumor necrosis factor-*α*.
